# Japanese Dentists

**Published:** 1885-07

**Authors:** 


### JAPANESE DENTISTS.

The following absurd statement first appeared in a metropolitan newspaper. It went the rounds of the large city dailies, then got into the literary weeklies, and finally became a stereotyped fixture in the patent-inside village regulators. As long as it was confined to the newspapers it was not worth while to contradict it. But lately it has been making its appearance in medical journals, and now it is time to enter a protest. We appeal, Gentlemen Medical Editors, to your compassion. Spare, Oh ! Spare us further perusals of this preposterous newspaper invention.

"In Japan the extraction of teeth has reached a degree of perfection absolutely unknown in France, and I might say in Europe or America, where they have good schools of dentistry. The Japanese dentists do not overwhelm their victims by a display of the instruments of torture with which our artists draw their client's bad teeth, not to mention the sound ones. It is with the thumb and index finger that the Japanese artist delicately withdraws you a molar or two. Naturally, great practice is required before arriving to such a degree of skillfulness. To obtain this the dentist pupil serves an apprenticeship to a master. For a long time he has to exercise himself in extracting bits of wood inserted in planks, loosely at first, but afterwards solidly fixed by hammer strokes in oak wood. When the pupil can, at a single trial, and without apparent effort, draw out one of these wooden teeth, any human jaw can be confided to his care, and no tooth, though fixed in a steel *aleoculus* (Alveolus) can resist him. A skillful Japanese operator can, in half a minute, and without removing his fingers from the victim's mouth, remove easily his half dozen teeth."

